# Health-Related Quality of Life among Patients with Coronary Artery Disease: A Post-Treatment Follow-Up Study in Iran

**DOI:** 10.1155/2012/973974

**Published:** 2012-06-10

**Authors:** Shahram Tofighi, Aliasghar Ahmad Kiadaliri, Jamil Sadeghifar, Mehdi Raadabadi, Jahanara Mamikhani

**Affiliations:** ^1^Health Management Research Centre, Baqiyatallah University of Medical Sciences, 1435814717 Tahran, Iran; ^2^Division of Health Economics, Department of Clinical Sciences-Malmö, Lund University, 20502 Malmö, Sweden; ^3^Health Economics & Management, Institute of Economic Research, Lund University, 22007 Lund, Sweden; ^4^Department of Health Management and Economics, School of Public Health, Tehran University of Medical Sciences, 141556447 Tehran, Iran; ^5^School of Health Services and Information Management, Tehran University of Medical Sciences, 1995614111 Tehran, Iran; ^6^Student Scientific Research Centre, Tehran University of Medical Sciences, Tehran, Iran; ^7^Behin Pooyan Hootan Research Institute, 1483895931 Tehran, Iran

## Abstract

*Objective.* To examine the changes in health-related quality of life (HRQoL) in patients with coronary artery disease (CAD) in terms of age, gender, and treatment strategy in Iran. *Methods and Materials.* Forty-nine patients responded to the Iranian version of the 36-item short form (SF-36) questionnaire to evaluate the HRQoL at first and third year after treatment. The paired and independent Wilcoxon rank-sum tests were used for within and between comparisons, respectively. Multivariate regression analysis was used to analyze the predictors of changes at HRQoL. *Results.* In general, during followup, the mental component summary scale improved, and the physical component summary scale declined. The results of multiple regression showed that the score at the first year post-treatment was the main predictor of HRQoL at follow up. Moreover, after adjusting for other covariates, receiving PTCA and being at older age were related to lower scores at followup, but these were not statistically significant in most cases. *Conclusion.* The HRQoL significantly changed from one to three years after treatment in patients with CAD. While, the physical health deteriorated during two-year follow up, mental health improved at the same time period. Generally, there were no significant differences at changes of HRQoL in terms of treatment, age, and gender.

## 1. Background

Health-related quality of life (HRQoL) shows a subjective and multidimensional concept that is composed of a range of domains, generally including physical, social, emotional, mental, and functional health [1].

It is believed that the traditional end points, which are mainly focused on the biologic and physiologic outcomes, may not capture the impact of the intervention on patients' HRQoL. As a result, a growing interest has appeared during the past decades for assessing and measuring the impact of diseases and their treatments on the patients' QoL, especially in long-term and chronic diseases [2]. Coronary artery disease (CAD) is one of these chronic diseases which impair the patient's functional capacity and quality of life [3, 4].

CAD is the first cause of death in Iran and accounts for 46% of all cause of deaths in the country [5]. The prevalence of the major risk factors for CAD is increasing in Iran [6] and it warns for more burden of disease in the future. In spite of this fact, little has been known about the predictors of HRQoL in patients with CAD in Iran. This provides a useful decision tool for policymakers when planning for provision of health services for these patients.

Previous studies have shown that treatment; gender, and age are some potential predictors of HRQoL in these patients [7–9]. The aim of current study was to evaluate the changes in HRQoL in patients with CAD, in terms of treatment type, age and gender, during the two-year followup, between one- to three-year post-treatment periods.

## 2. Method and Material

In a prospective cohort design, among patients with CAD who underwent PTCA or CABG between March 1st 2007 and September 31st 2008 in two hospitals in Tehran, 49 consecutive patients who met inclusion criteria were included in the study. Inclusion criteria were (a) living in city of Tehran, (b) having two or three vessel coronary artery diseases, (c) no preposing disease, and (d) consent to participate in the study. A culturally comparable questionnaire of the Short Form Health Survey (SF-36) [10] has been developed, translated, and validated previously in the Iranian population [11]. The current study utilized this Iranian version of the SF-36 in order to evaluate the HRQoL of the patients with CAD. The questionnaire includes multi-item scales to assess the eight dimensions of wellness: physical functioning, role limitations due to physical health problems, bodily pain, general health perceptions, social functioning, vitality, energy or fatigue, and role limitations due to emotional problems. In addition, two summary scores are calculated using these eight scales: physical component summary (PCS) and mental component summary (MCS).

 Each of the subscales is scored on a scale of 0–100, with higher scores indicating better HRQoL. The summary components were calculated using the mean value of each score for the Iranian population [11] and the coefficients from a US study [12].

This questionnaire has been extensively used to examine the HRQoL in cardiac patients [8, 13, 14]. Moreover, a recent study in Iran has shown that SF-36 is a valid and reliable tool for assessing the HRQoL in patients with CAD [15].

The first round post-treatment interviews were done between January and May 2009. Patients were followed to April and May 2011 when they were interviewed for the second time. Both interview rounds were conducted by face to face contact.

### 2.1. Statistical Analysis

Two types of statistical analyses were applied, univariate and multivariate analysis. In univariate analysis, due to skewness in HRQoL data, the paired Wilcoxon rank-sum test was used to detect any significant changes in HRQoL during the followup. Moreover, independent Wilcoxon rank-sum test was used to test if there is any significant difference in HRQoL in terms of treatment, age, and gender. Finally, multiple regression analysis was used to examine the main predictors of changes in HRQoL during the study period. Data were analyzed using STATA statistical package, version 11 [16].

## 3. Results

Altogether, 49 patients participated in the current study, 24 of whom underwent the CABG and 25 received PTCA.  Table 1 shows the characteristics of the patients at the time of first interview in both treatment groups. Mean age was 51.75 (±8.46) and 49.28 (6.53) in CABG and PTCA groups, respectively. On average, patients in both groups received the treatment about one year prior to the first round interview (*P* = 0.219).

The median of followup was 25.6 months.  Table 2 shows the SF-36 subscales scores in the total sample in the first round and follow-up interviews. There was positive correlation between physical and mental components summary scales in two interviews (*r*_1_ = 0.171; *P* = 0.24 and *r*_2_ = 0.299; *P* = 0.04). In general, physical functioning, role-emotional, mental health, and mental components summary scales improved significantly during the followup. On the other hand, bodily pain, social functioning, and physical components summary scales deteriorated in the same period. The greatest improvement (41.69%) and decline (12.10%) were seen in mental health and social functioning, respectively.

Table 3 shows the results of univariate analysis in terms of treatment. Patients experienced significant improvements in mental health and mental component summary scales during the followup. However, the level of physical component summary decreased at the same period. There were no significant differences in changes of HRQoL during the followup between two treatment groups (Table 3, last column). 

The results of univariate analysis for gender groups have been shown in Table 4. Males and females experienced significant improvements in 3 out of 10 scales during the followup. On the other hand, four and one scales significantly deteriorated during the followup in both males and females, respectively. Except for role-physical scale, the directions of changes in men were similar to women. Only the change in the physical functioning score was significantly different between males and females (Table 4, last column). 

Table 5 presents the HRQoL experiences in terms of age groups. The median age at the baseline was used as a cut-off point. Mental health and mental component summary scores increased between the two interview periods in both age groups, while the social functioning declined. The higher number of scales deteriorated for older patients compared with younger ones (4 versus 1). Except for role-physical scale, the directions of changes in scales were similar in the two groups. Older patients experienced higher changes in role-physical scale than younger ones (Table 5, last column). 

The results of multivariate analysis have been presented in Table 6. Patients with the higher score at the first year post-treatment had a higher score at the follow-up. After control for other covariates, older patients, on average, had lower scores at role-physical and role-emotional scales than younger ones in the followup. There were no significant differences in HRQoL between CABG and PTCA groups, after control for other covariates. The follow-up score of social functioning was higher for males than females. Variations explained by the regression models ranged between 22% in role-physical scale and 48% in vitality scale. 

## 4. Discussion

The changes of HRQoL are considered as an important outcome in treatment guidelines in different medical fields. In the current study the changes of HRQoL in the patients with CAD during the period of one to three years after treatment were examined to see if type of treatment; age and gender can explain the differences in HRQoL in these patients. 

During the followup, based on mental and physical component summary scales, the mental health significantly improved while the physical health deteriorated. These changes in mental and physical health were in line with previous studies [17, 18]. 

It should be noted that physical functioning was improved significantly during the followup, but physical health as measured by physical component summary declined. In all age, gender, and treatment groups, the greatest improvements were seen in the mental health scale. Generally, the size and direction of changes in HRQoL between age, gender, and treatment groups were similar. 

In terms of treatment groups (CABG versus PTCA), although both groups experienced significant changes in HRQoL during the followup, there were no significant differences in these changes between the two groups. The significant changes in HRQoL after treatment in both treatment groups were reported in previous studies [3, 8]. In addition, previous studies have shown that the differences in HRQoL between CABG and PTCA become smaller over time, and the differences are not significant during the followup [19–21]. 

The number of the SF-36 scales which significantly changed during the followup was higher for males than females (7 versus 4). Generally, male had higher scores at the followup than females, after control for other covariates; but it was only statistically significant for social functioning. Previous studies have shown that, generally, the level of HRQoL for males is higher than females in patients with CAD [7, 22]. 

Both younger and older patients experienced significant changes in HRQoL from one- to three-year post-treatment period. After adjusting for other covariates, older patients had lower scores in role-physical and role-emotional scales at the followup than younger patients. In a previous study, Höfer et al. [23] found that older patients with CAD had a lower level of ability to perform physical tasks. Worse HRQoL for older patients with CAD was also reported by Unsar et al. [22]. 

The scores at the first interview round were the main predictors of the follow-up scores. Patients with a higher level of HRQoL at one-year post-treatment experienced a higher level of HRQoL over three years after the treatment. Hence, the effects of treatments on HRQoL during first year after treatment can be used as a measure for long-term effects of treatment in patients with CAD. 

The findings of current study expand the body of knowledge about HRQoL among the patients who suffer from the leading cause of deaths in Iran. However, the results of this study should be interpreted in light of a number of limitations. The number of patients in the study was small and it may affect the produced results. Although, nonrandomization design raises the chance of biases in our study, it reflects the routine practice in the country. Moreover, SF-36 is a general instrument to measure the HRQoL and may have not captured the aspects of HRQoL which is specific to CAD. Using the disease-specific instruments and comparing the results with general instruments is an agenda for future research. Furthermore, the cultural adjustment in SF-36 for using in the Iranian population may constrain the direct comparison of the current study with studies in other countries and hence limit the generalizability of its results to other jurisdictions. 

## 5. Conclusion 

The HRQoL significantly changed from one to three years after the treatment in the patients with CAD. While the physical health deteriorated during the two-year followup, mental health improved at the same time. Generally, there were no significant differences in HRQoL changes in terms of treatment strategy, age, and gender. HRQoL at the first year after treatment is an important predictor of HRQoL over long-term followup.

## Figures and Tables

**Table 1:** Patients' characteristics at the first round interview (first year post-treatment).

Variable	CABG	PTCA
No. of patients	24	25
Median time since intervention (months)	10.94	8.84
Male (%)	66.66	80.00
Age (mean ± SD)	51.75 (8.46)	49.28 (6.53)
Age-groups (years old)		
<50 (frequency)	12	11
50–60 (frequency)	9	12
60 and older	3	2
Covered by health insurance plan (%)	100.00	100.00

**Table 2:** Comparison of the HRQoL scores at the first and third year post-treatment.

	First year (SD)	Third year (SD)	Change (%)	*P*-value
Physical functioning	79.47 (26.96)	84.32 (23.91)	6.10	0.036
Role-physical	80.61 (30.34)	75.51 (32.07)	−6.33	0.302
Bodily pain	80.29 (24.88)	73.06 (26.62)	−9.00	0.016
General health	63.39 (20.45)	58.07 (23.97)	−8.39	0.063
Vitality	61.52 (22.81)	66.78 (18.13)	8.55	0.229
Social functioning	80.94 (27.32)	71.15 (25.52)	−12.10	<0.001
Role-emotional	75.53 (41.82)	87.24 (30.12)	15.50	0.007
Mental health	54.81 (25.93)	77.66 (16.94)	41.69	<0.001
Physical component summary	50.35 (9.86)	46.07 (10.51)	−8.50	0.001
Mental component summary	48.01 (12.93)	54.99 (8.58)	14.54	<0.001

**Table 3:** The HRQoL scores at the first and third year post-treatment in CABG and PTCA treatment groups.

	CABG (*n* = 24)			PTCA (*n* = 25)			Between groups comparison
	Baseline (SD)	Followup (SD)	*P*-value	Baseline (SD)	Followup (SD)	*P*-value	*P*-value
Physical functioning	79.41 (21.80)	84.21 (19.01)	0.174	79.53 (31.59)	84.42 (28.24)	0.078	0.716
Role-physical	80.21 (31.26)	73.96 (31.69)	0.450	81.00 (30.00)	77.00 (33.09)	0.529	0.692
Bodily Pain	76.95 (22.66)	73.39 (25.31)	0.333	83.50 (26.90)	72.75 (28.35)	0.027	0.120
General health	65.89 (18.34)	64.02 (21.02)	0.797	61.00 (22.41)	52.36 (25.62)	0.042	0.101
Vitality	61.61 (23.15)	70.60 (15.32)	0.056	61.43 (22.96)	63.11 (20.09)	0.925	0.134
Social functioning	87.50 (22.42)	73.95 (20.68)	0.003	74.64 (30.43)	68.46 (29.62)	0.082	0.285
Role-emotional	86.13 (33.92)	93.04 (19.68)	0.307	65.36 (46.63)	81.68 (37.11)	0.008	0.158
Mental health	54.46 (23.91)	81.58 (9.94)	<0.001	55.14 (28.24)	73.89 (21.19)	0.002	0.215
Physical component summary	49.72 (9.30)	45.98 (9.90)	0.032	50.96 (10.52)	46.16 (11.27)	0.015	0.631
Mental component summary	49.89 (11.59)	57.49 (4.25)	0.002	46.20 (14.10)	52.59 (10.85)	0.020	0.535

**Table 4:** The HRQoL scores at the first and third year post-treatment in based on gender.

	Male (*n* = 36)			Female (*n* = 13)			Between groups comparison
	Baseline (SD)	Followup (SD)	*P*-value	Baseline (SD)	Followup (SD)	*P*-value	*P*-value
Physical functioning	85.95 (21.48)	87.43 (22.20)	0.482	61.54 (32.98)	75.71 (27.22)	0.008	0.016
Role-physical	88.89 (20.22)	78.47 (26.83)	0.096	57.69 (41.31)	67.31 (43.76)	0.571	0.207
Bodily pain	85.12 (22.24)	76.08 (22.99)	0.003	66.92 (27.72)	64.71 (34.51)	0.859	0.112
General health	67.19 (20.18)	61.36 (20.08)	0.020	52.88 (17.97)	48.95 (31.61)	0.753	0.303
Vitality	64.88 (21.35)	69.29 (14.64)	0.315	52.20 (24.98)	59.83 (24.86)	0.553	0.910
Social functioning	83.76 (26.63)	75.80 (21.47)	0.013	73.12 (28.75)	58.27 (31.87)	0.019	0.355
Role-emotional	76.86 (42.03)	88.19 (27.51)	0.033	71.85 (42.69)	84.62 (37.55)	0.084	0.698
Mental health	58.13 (26.90)	79.24 (12.95)	<0.001	45.60 (21.34)	73.28 (25.10)	0.002	0.292
Physical component summary	53.09 (7.61)	47.56 (9.21)	<0.001	42.77 (11.63)	41.97 (13.02)	0.807	0.160
Mental component summary	48.61 (13.71)	55.79 (7.51)	0.001	46.34 (10.77)	52.78 (11.09)	0.028	0.651

**Table 5:** The HRQoL scores at the first and third year post-treatment based on the age groups.

	<50 years old (*n* = 23)			≥50 years old (*n* = 26)			Between groups comparison
	Baseline (SD)	Followup (SD)	*P*-value	Baseline (SD)	Followup (SD)	*P*-value	*P*-value
Physical functioning	91.30 (14.06)	94.05 (11.37)	0.105	69.00 (31.28)	75.71 (28.64)	0.138	0.663
Role-physical	83.70 (25.68)	88.04 (24.85)	0.420	77.88 (34.15)	64.42 (34.04)	0.022	0.045
Bodily pain	85.11 (22.27)	82.55 (15.30)	0224	76.03 (26.68)	64.66 (31.60)	0.027	0.618
General health	66.85 (22.96)	61.26 (17.81)	0.153	60.34 (17.85)	55.24 (28.39)	0.286	0.405
Vitality	68.94 (19.09)	72.22 (13.40)	0.891	54.95 (24.14)	61.97 (20.53)	0.127	0.237
Social functioning	89.17 (20.34)	79.91 (13.24)	0.008	73.65 (30.84)	63.40 (31.04)	0.023	0.746
Role-emotional	79.74 (38.57)	96.74 (13.99)	0.055	71.81 (44.91)	78.85 (37.59)	0.046	0.467
Mental health	65.84 (21.75)	84.21 (9.52)	0.001	45.05 (25.78)	71.86 (19.91)	<0.001	0.109
Physical component summary	52.63 (5.81)	49.63 (6.59)	0.114	48.33 (12.16)	42.93 (12.33)	0.003	0.155
Mental component summary	51.72 (10.28)	57.59 (3.74)	0.029	44.72 (14.29)	52.70 (10.84)	0.001	0.271

**Table 6:** Results of multiple regression analyses*.

	SF-36 subscales									
	Physical functioning	Role-physical	Bodily pain	General health	Vitality	Social functioning	Role-emotional	Mental health	Physical component summary	Mental component summary
Score at first-year **	0.488^a^	0.268	0.506^a^	0.340^c^	0.382^a^	0.488^a^	0.433^a^	0.184^c^	0.557^a^	0.298^a^
≥50 years old	−8.281	−22.916^b^	−13.620^c^	−2.815	−4.388	−9.927	−14.748^b^	−8.947^c^	−4.483^c^	−2.842
PTCA	−0.961	3.257	−3.558	−11.313	−8.154^c^	−2.176	−1.711	−8.718^c^	−0.552	−4.222^c^
Male	2.289	2.645	2.110	10.229	6.366	15.132^b^	1.102	5.513	0.322	3.047
Interval_1**	0.039	0.016	0.001	0.006	0.005	0.044^c^	−0.000	0.017	0.008	0.004
Interval_2**	−0.093	−0.013	0.039	−0.043	−0.019	−0.090	0.032	0.003	−0.016	0.007
Constant	87.521^a^	88.898	80.553^a^	57.821^a^	68.591^a^	66.407^a^	95.134^a^	82.803^a^	48.498^a^	56.416^a^
*R*-squared	0.462	0.220	0.333	0.225	0.479	0.462	0.463	0.311	0.373	0.397

*The scores at followup were regressed on covariates.

a,  b  and  c denote the significance level at the 1, 5, and 10%, respectively.

**These covariates transformed as mean centering.
